# Nucleotide metabolic abnormalities in post-COVID-19 condition and type 2 diabetes mellitus patients and their association with endocrine dysfunction

**DOI:** 10.1515/med-2025-1221

**Published:** 2025-10-07

**Authors:** Yalei Fan, Xiaomin Xie, Guirong Bai, Wenrui Ji, Yanting He, Li Zhang, Haiyan Zhou, Ling Li, Huan Li, Dan Qiang

**Affiliations:** The Second Clinical Medical School of Ningxia Medical University, Yinchuan, 750001, China; Department of Endocrinology, The Second Affiliated Hospital of Ningxia Medical University, Liqun West Street 2, Xingqing District, Yinchuan, Ningxia Hui Autonomous Region, 750001, China; Department of Endocrinology, The Second Affiliated Hospital of Ningxia Medical University, Yinchuan, Ningxia Hui Autonomous Region, 750001, China

**Keywords:** post-COVID-19 condition, type 2 diabetes mellitus, UPLC–MS/MS, metabolites, nucleotide metabolism

## Abstract

COVID-19 virus infection can cause disorders of the endocrine system. The aim of this study was to characterize the alterations of nucleotide metabolomic patterns in patients with post-COVID-19 condition (PCC). The study population included 18 patients with PCC alone (PCC-A), 31 patients with PCC combined with type 2 diabetes mellitus (PCC-DM), 20 healthy volunteers (HV), and 20 patients with type 2 diabetes mellitus (DM). Ultraperformance liquid chromatography–mass spectrometry was conducted on plasma metabolites. A total of 116, 178, and 163 differential metabolites were identified in PCC-DM vs PCC-A, PCC-A vs HV groups, and PCC-DM vs DM groups, respectively. Adenine was significantly down-regulated, and purine, thymine, and uracil were significantly up-regulated in the PCC-A group compared with the HV group, and the same results were observed in the PCC-DM group compared with the DM group. Differential metabolites were mainly involved in nucleotide metabolism, especially pyrimidine metabolism in PCC patients. After the arginine stimulation test, cortisol and adrenocorticotropic hormone secretion were reduced in PCC patients. In conclusion, the nucleotide *de novo* synthesis pathway and the remedial synthesis pathway are seriously damaged in patients with PCC-A, especially in patients with PCC-DM, which leads to the disorder and imbalance of the body cell metabolism pathway.

## 1 Introduction

Since the global epidemic of coronavirus disease 2019 (COVID-19) began in 2019, some patients have a series of long-term clinical symptoms involving multiple systems and organs of the body after treatment, that is, long COVID, also called post-COVID-19 condition (PCC) [1]. PCC is defined as 3 months after COVID-19 infection; at least 10% of patients develop long-term symptoms, including fatigue, intermittent headache, cough, dyspnea, decreased smell, and muscle pain, and may also have memory loss, sleep disturbance, chest pain or tightness, heart palpitations, depression and anxiety, nausea, diarrhea, and rash. These symptoms reflect the chronic damage of multi-system organs, not only damaging the nervous system, digestive system, reproductive system, and motor system, but also causing endocrine system disorders [2,3,4,5]. Currently, the main hypotheses for the pathogenesis of PCC include changes in the immune system, chronic inflammation, endothelial dysfunction, microthrombosis, mitochondrial dysfunction, metabolic abnormalities, activation of chronic viral infection, microbiome dysregulation, and unresolved tissue damage [6]. PCC affects the quality of life of patients, increases the cost of social health care, and causes a huge burden to families and society. Therefore, it is very important to study the long-term health status and dynamic changes of patients with PCC.

COVID-19 is closely associated with endocrine function and can affect the endocrine functions of the pituitary gland, thyroid, adrenal glands, gonads, and pancreas [7]. Studies have confirmed that COVID-19 can invade many parts of the brain, including the hypothalamus and pituitary gland, and the COVID-19 genome is present in the cerebrospinal fluid [8]. Adrenocorticotropic hormone (ACTH) and cortisol (F) exert a crucial role in the hypothalamic–pituitary–adrenal axis [9]. ACTH secretion is significantly decreased in COVID-19 patients compared to healthy individuals [10]. ACTH and growth hormone decreased in the anterior pituitary cells of SARS patients [11]. In addition, the levels of F were discovered to be diminished in COVID-19 patients compared with the controls [12]. The mechanisms by which COVID-19 affects endocrine function include direct viral damage, endothelial dysfunction secondary to SARS-CoV-2-induced endotheliitis, and immune-mediated organ damage caused by uncontrolled cytokine release [13]. SARS-CoV-2 could induce endothelial dysfunction via regulating ACE2, AXL, and L-SIGN expression [14]. A recent study has demonstrated that SARS-CoV-2 led to enhanced caspase-3 cleavage and apoptotic cell death in endothelial cells [15]. The cytokine storm triggered by severe COVID-19 (e.g., IL-6, IL-1β, TNF-α, and MCP-1) was a good predictor of the severity of COVID-19, and it also intensified endothelial cell damage [16]. However, the underlying mechanism of endocrine hormone disruption in PCC patients is not fully understood.

In recent years, metabolomics has emerged as a vital field. It delves deeply into metabolic processes and excavates potential biomarkers. As a result, it has become a powerful tool for determining metabolic perturbations across a wide range of diseases [17,18]. The metabolomic techniques mainly include liquid chromatography–mass spectrometry (LC–MS), gas chromatography–mass spectrometry (GC–MS), and ultra-performance liquid chromatography–tandem mass spectrometry (UPLC–MS/MS) [19,20,21]. López‑Hernández et al. found that patients with long COVID-19 exhibited mitochondrial dysfunction, redox state imbalance, and lipid metabolism dysregulation through LC–MS/MS analysis [22]. Saito et al. discovered that amino acid metabolomic abnormalities occurred in long COVID patients by LC–MS analysis [23]. Metabolomic studies in PCC patients have highlighted alterations in lipid metabolism, amino acid metabolism, and the tricarboxylic acid cycle [22,24,25]. Currently, the persistence of SARS-CoV-2 was significantly associated with PCC [26]. The latest research indicates that the proteins expressed by SARS-CoV-2 are involved in cellular nucleotide metabolism [27]. However, it remains unclear whether patients with PCC exhibited abnormal nucleotide metabolism and whether such disturbances are associated with endocrine dysfunction.

In this study, we deciphered the differential metabolites and nucleotide metabolism in the subjects with or without PCC using UPLC–MS/MS. We also investigated the relationship between differential metabolites and ACTH and F in patients with PCC to explore new mechanisms of endocrine hormone disorders in PCC patients. This study will provide potential biomarkers and therapeutic targets for PCC patients.

## 2 Methods

### 2.1 Research objects

A total of 49 PCC patients aged 18–75 years old, including 28 females and 21 males, were enrolled in the Department of Endocrinology, the Second Affiliated Hospital of Ningxia Medical University from September 2023 to November 2023. Among the PCC patients, 18 patients were diagnosed with PCC alone (PCC-A) and 31 patients were diagnosed with PCC combined with type 2 diabetes mellitus (PCC-DM). The control group included 40 subjects aged 18–75 years old, including 20 females and 20 males, who had not been infected with SARS-CoV-2 in the medical examination center of our hospital from September to November 2021. Among the 40 controls, 20 cases were healthy volunteers (HV) and 20 cases were type 2 diabetes mellitus (DM). The diagnostic criteria for PCC and DM patients are as follows: all patients with DM met the 2019 American ADA diagnostic criteria [28] and all patients with PCC met the PCC diagnostic criteria [29]. PCC-DM patients meet the above two diagnostic criteria. Exclusion criteria are as follows: (a) patients with type 1 diabetes and other specific types of diabetes; (b) patients with acute and chronic complications of diabetes; (c) patients with chronic kidney or liver disease or cancer; (d) patients with secondary hypertension; (e) patients with anemia and history of severe cardiovascular or cerebrovascular diseases or tumors; and (f) the woman is not pregnant or lactating.

### 2.2 Blood index detection

Peripheral venous blood was collected after fasting 8–12 h. Fasting blood glucose, alanine aminotransferase (ALT), aspartate aminotransferase (AST), total protein (TP), albumin (ALB), triglyceride (TG), total cholesterol (TC), high-density lipoprotein (HDL), low-density lipoprotein (LDL), creatinine (Cr), and uric acid (UA) were measured using the automatic biochemical analyzer AU5821. Plasma cortisol (F) and ACTH were detected by electrochemiluminescence assay using the Cobas 6000 automatic biochemical immunoanalyzer. The difference of F and ACTH at 0, 30, 60, 90, and 120 min after intravenous infusion of arginine was calculated. Plasma samples from all subjects were stored in a −80°C refrigerator for subsequent metabolomics.

### 2.3 Arginine excitation test

Fasting blood samples were collected from PCC patients at 8:00 am and preserved in an ice bath. PCC patients were given an intravenous infusion of arginine at a dosage of 0.5 g/kg (the maximum dosage was 30 g), and the infusion was finished within 30 min. Blood samples were taken before and after arginine injection at –30 (fasting), 0 (the end of arginine infusion), 30, 60, 90, and 120 min and stored in an ice bath. F and ACTH were detected at all time periods.

### 2.4 Metabolites extraction

Plasma samples were subjected to vacuum freeze-drying in a lyophilizer, followed by grinding into powder using a grinder. A weight of 100 mg of powder was then dissolved in 1.2 mL of 70% methanol extract. The mixture was vortexed once every 30 min, with each vortexing session lasting 30 s, for a total of six times. The sample was subsequently refrigerated at 4°C overnight. After centrifugation, the supernatant was carefully aspirated, filtered through a microporous filter membrane, and stored in an injection bottle for subsequent UPLC–MS/MS analysis.

### 2.5 UPLC conditions

Chromatographic separation was conducted on a column Agilent SB-C18 (2.1 mm × 100 mm, 1.8 µm). The mobile phase included 0.1% formic acid in ultra-pure water (phase A) and acetonitrile (phase B). Elution for phase B started at 5%, increased to 95% within 9 min, then returned to 5% in 10 min, and balanced at 5% for 14 min. A total of 4 μL samples was injected into the detector and separated on a column maintained at 40°C with a flow rate of 0.35 mL/min.

### 2.6 LC–MS/MS analysis

Linear ion trap and triple quadrupole (QQQ) scans were obtained on a triple quadrupole-linear ion trap mass spectrometer (QTRAP), AB6500 QTRAP LC–MS/MS System. This system is equipped with an ESI Turbo Ion-Spray interface and is capable of operating in both positive and negative ion modes and controlled by Analyst 1.6.3 software (Sciex). The ESI source operation parameters were set as follows: the source temperature was set to 550°C, and the ion spray voltage was set to 5,500 V for the positive mode and −4,500 V for the negative mode. The nebulizer gas (GSI), heater gas (GSII), and curtain gas were set at 50, 60, and 25 psi, respectively. Collision-induced ionization parameter was set to high. The instrument was tuned and calibrated with 10 and 100 μmol/L polypropylene glycol solution in QQQ and LIT modes, respectively. The QQQ scan uses the multiple reaction monitoring (MRM) mode and sets the collision gas (nitrogen) to medium. A specific set of MRM ion pairs is monitored at each period based on the metabolites elution during each period. Based on a self-constructed database, substance identification is carried out according to secondary spectral information. During analysis, isotopic signals, duplicate signals containing K^+^ ions, Na^+^ ions, \[{\text{NH}}_{4}^{+}]\] ions, as well as duplicate signals that are fragments of larger molecular weight substances, are removed. Metabolite quantification was accomplished using the MRM mode of a triple quadrupole mass spectrometer. In the MRM mode, the quadrupole first screened for the precursor ions of the target substances, excluding ions corresponding to other molecular weights to initially eliminate interference. After ionization induced by the impact chamber, the precursor ions were fractured to form many fragments, and then, a characteristic fragment ion was selected by filtering through the triple quadrupole. After obtaining the metabolite mass spectrometry data from different samples, the peak areas of all substance mass spectrometry peaks were integrated, and the peak integrations of the same metabolite across different samples were corrected. The mass spectrometry data were processed using the software Analyst 1.6.3.

Principal component analysis (PCA) and Spearman correlation analysis were employed to assess the repeatability of samples within each group, as well as the quality control samples, ensuring the reliability and consistency of the data. The identified compounds were cross-referenced with the KEGG, HMDB, and Lipidmaps databases to obtain classification and pathway information. A *t*-test was conducted to determine the significance of the differences for each compound. Metabolites were listed in Tables S1–S3. Based on the orthogonal partial least squares discriminant analysis (OPLS-DA) results, the variable importance in projection (VIP) from the obtained multivariate OPLS-DA model can be used for an initial screening of differential metabolites. Differential metabolites were screened based on the criteria of fold change ≥1, *p*-value <0.05, and VIP ≥1. Fisher’s exact test was utilized to compute *p*-values based on the method of Benjamini and Hochberg for multiple testing corrections. Pathway analysis was conducted by utilizing the KEGG pathway database through the MetaboAnalyst 5.0 online software.

### 2.7 Statistical analysis

SPSS 26.0 (IBM, USA) software was used for statistical analysis, and the data were expressed as mean ± standard deviation. A *t*-test was used for comparison between the two groups. Correlations were analyzed by Pearson correlation analysis. The difference of *p* < 0.05 was considered statistically significant.


**Ethical approval:** This study was approved by the Ethics Committee of The Second Affiliated Hospital of Ningxia Medical University.
**Informed consent:** All subjects received informed consent.

## 3 Results

### 3.1 The biochemical and hormonal level changes in PCC patients

The biochemical and hormonal level changes in patients with PCC-A and patients with PCC-DM were analyzed. There was no significant difference in weakness, edema, hypomnesis, muscular soreness, sleep disorders, depression/anxiety, erythra, headache, cough, chest distress, palpitation, nausea, constipation, diarrhea, and hyposmia/hypogeusia between PCC patients and patients with PCC-DM (Table 1). Subsequently, the changes in biochemical indexes and hormone levels were analyzed. Compared with PCC patients, the levels of FBG and Hb1c in patients with PCC-DM were significantly increased (Table 2). However, no significant difference was observed in BMI, ALT, AST, TP, ALB, TG, TC, LDL, Cr, and UA levels between the two groups (Table 2). F and ATCH levels were detected at baseline, 0, 30, 60, and 90 min after the arginine stimulation test. As shown in Table 3, F and ATCH levels were gradually decreased as time went on in patients with PCC-A and patients with PCC-DM. There was no significant difference found in F level and ACTH level at any time between the two groups. Overall, ACTH and F cannot secrete normally after stress in PCC patients.

**Table 1:** Main clinical manifestations of PCC patients

	PCC-A (*N* = 18)	PCC-DM (*N* = 31)	\[{\chi }^{2}]\]	*P*
Weakness	8/18 (44.4%)	12/31 (38.7%)	0.155	0.694
Edema	2/18 (11.1%)	5/31 (16.1%)	0.234	0.628
Hypomnesis	9/18 (50.0%)	14/31 (45.2%)	0.107	0.744
Muscular soreness	9/18 (50.0%)	14/31 (45.2%)	0.107	0.744
Sleep disorders	9/18 (50.0%)	14/31 (45.2%)	0.107	0.744
Depression/anxiety	10/18 (55.5%)	14/31 (45.2%)	0.492	0.483
Erythra	0/18 (0.0%)	1/31 (3.2%)	0.593	0.441
Headache	3/18 (16.7%)	6/31 (19.4%)	0.055	0.815
Cough	2/18 (11.1%)	2/31 (6.5%)	0.330	0.566
Chest distress	1/18 (5.5%)	6/31 (19.4%)	1.771	0.183
Palpitation	1/18 (5.5%)	2/31 (6.5%)	0.02	0.900
Nausea	2/18 (11.1%)	3/31 (9.7%)	0.03	0.873
Constipation	1/18 (5.5%)	3/31 (9.7%)	0.258	0.611
Diarrhea	0/18	0/31	—	—
Hyposmia/Hypogeusia	0/18	0/31	—	—

PCC-A: PCC alone. PCC-DM: PCC combined with type 2 diabetes mellitus.

**Table 2:** Biochemical changes of PCC patients

	PCC-A (*N* = 18)	PCC-DM (*N* = 31)	*T*	*P*
BMI (kg/m^2^)	25.83 ± 3.69	25.29 ± 3.85	0.475	0.637
FBG (mmol/L)	4.96 ± 0.62	7.59 ± 3.18	−3.456	0.001
Hb1c (%)	5.45 ± 0.31	8.26 ± 2.33	−5.064	0.000
ALT (U/L)	19.42 ± 11.29	25.81 ± 19	−1.296	0.201
AST (U/L)	19.74 ± 5.75	22.03 ± 9.47	−0.93	0.357
TP (g/L)	67.6 ± 5.07	66.68 ± 7.73	0.451	0.654
ALB (g/L)	40.99 ± 1.77	40.14 ± 5.43	0.64	0.526
TG (mmol/L)	1.4 ± 0.68	2.13 ± 1.81	−1.647	0.106
TC (mmol/L)	4.19 ± 1.05	4.48 ± 1.24	−0.836	0.407
LDL (mmol/L)	2.08 ± 0.62	2.34 ± 0.77	−1.212	0.232
Cr (umol/L)	61.68 ± 8.45	66.18 ± 14.74	−1.184	0.242
UA (mmol/L)	346.87 ± 92.88	337.39 ± 103.48	0.321	0.75

PCC-A: PCC alone. PCC-DM: PCC combined with type 2 diabetes mellitus. BMI: body mass index. FBG: fasting blood glucose. Hb1c: hoglobin1c. ALT: alanine transaminase. AST: aspartate aminotransferase. TP: total protein. ALB: albumin. TG: triglyceride. TC: total choiesterol. LDL: low-density lipoprotein. Cr: creatinine. UA: uric acid.

**Table 3:** Hormone level changes of PCC patients undergoing the arginine stimulation test

	PCC-A (*N* = 18)	PCC-DM (*N* = 31)	*T*	*P*
F baseline (nmol/L)	2.46 ± 0.27	2.47 ± 0.38	−0.07	0.944
F 0 min (nmol/L)	2.46 ± 0.23	2.4 ± 0.35	0.584	0.562
F 30 min (nmol/L)	2.43 ± 0.18	2.35 ± 0.35	0.903	0.371
F 60 min (nmol/L)	2.39 ± 0.18	2.31 ± 0.34	0.981	0.332
F 90 min (nmol/L)	2.33 ± 0.23	2.29 ± 0.33	0.377	0.708
F 0 min growth value (nmol/L)	−0.01 ± 0.19	−0.07 ± 0.13	1.37	0.177
F 30 min growth value (nmol/L)	−0.03 ± 0.22	−0.12 ± 0.14	1.695	0.097
F 60 min growth value (nmol/L)	−0.07 ± 0.25	−0.16 ± 0.15	1.595	0.117
F 90 min growth value (nmol/L)	−0.14 ± 0.27	−0.18 ± 0.16	0.661	0.512
ACTH baseline (nmol/L)	0.73 ± 0.28	0.71 ± 0.29	0.314	0.755
ACTH 0 min (nmol/L)	0.76 ± 0.34	0.59 ± 0.3	1.724	0.091
ACTH 30 min (nmol/L)	0.64 ± 0.27	0.52 ± 0.28	1.487	0.144
ACTH 60 min (nmol/L)	0.58 ± 0.3	0.52 ± 0.23	0.878	0.384
ACTH 90 min (nmol/L)	0.44 ± 0.25	0.53 ± 0.21	−1.463	0.15
ACTH 0 min growth value (nmol/L)	0.02 ± 0.28	−0.11 ± 0.21	1.923	0.061
ACTH 30 min growth value (nmol/L)	−0.09 ± 0.31	−0.18 ± 0.23	1.214	0.231
ACTH 60 min growth value (nmol/L)	−0.15 ± 0.42	−0.19 ± 0.24	0.422	0.675
ACTH 90 min growth value (nmol/L)	−0.3 ± 0.34	−0.17 ± 0.22	−1.541	0.13

PCC-A: PCC alone. PCC-DM: PCC combined with type 2 diabetes mellitus.

### 3.2 Metabolomics multivariate analysis in PCC patients with DM

**Figure 1:**
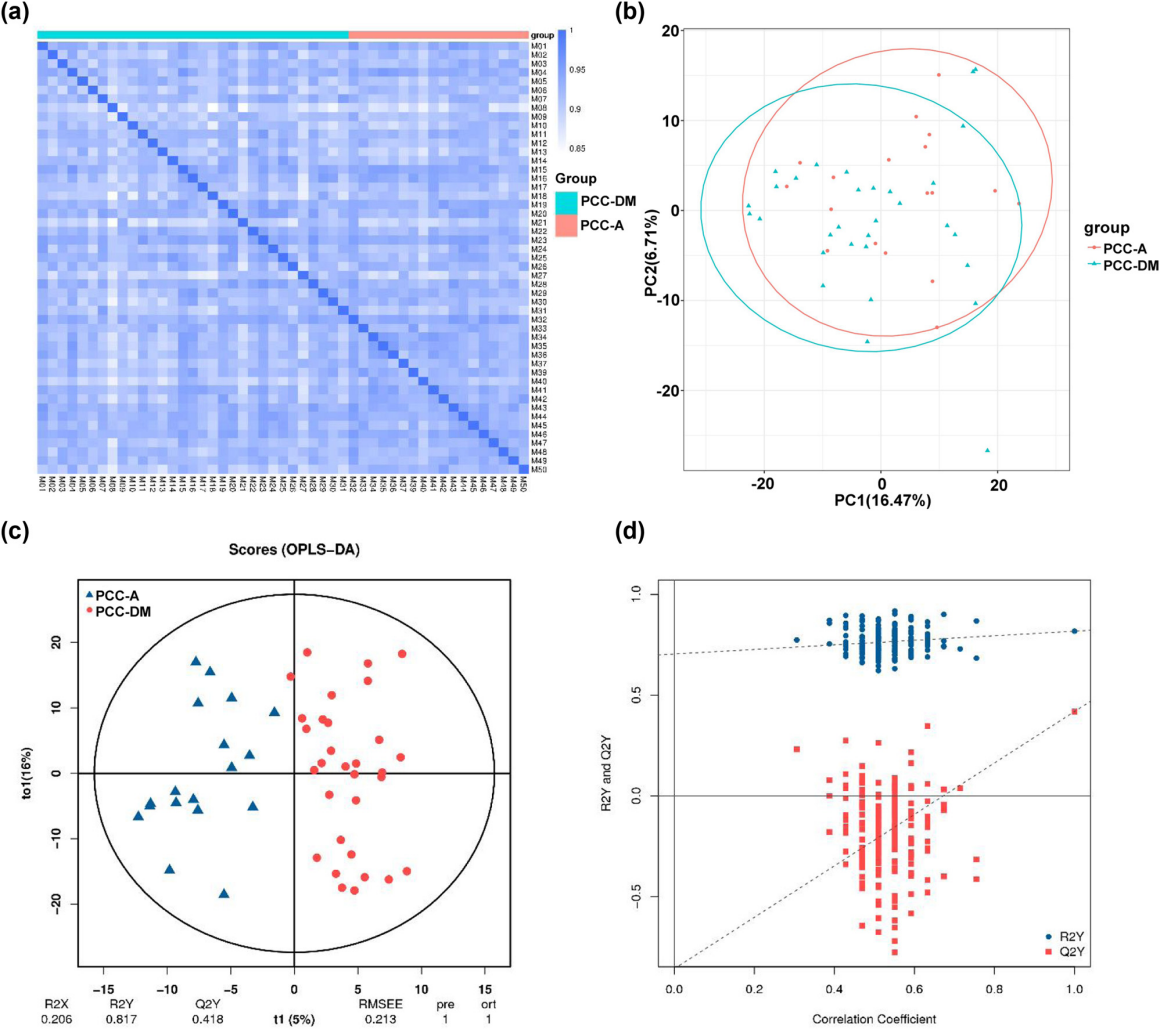
Multivariate analysis of metabolites from patients with PCC-A and patients with PCC-DM. (a) Correlation analysis between samples. (b) PCA score plot based on the metabolites in PCC-DM and PCC-A groups. (c) OPLS-DA plot based on the metabolites in PCC-DM and PCC-A groups. (d) OPLS-DA permutation test in PCC-DM and PCC-A groups. PCC-A: PCC alone. PCC-DM: PCC combined with type 2 diabetes mellitus.

**Figure 2:**
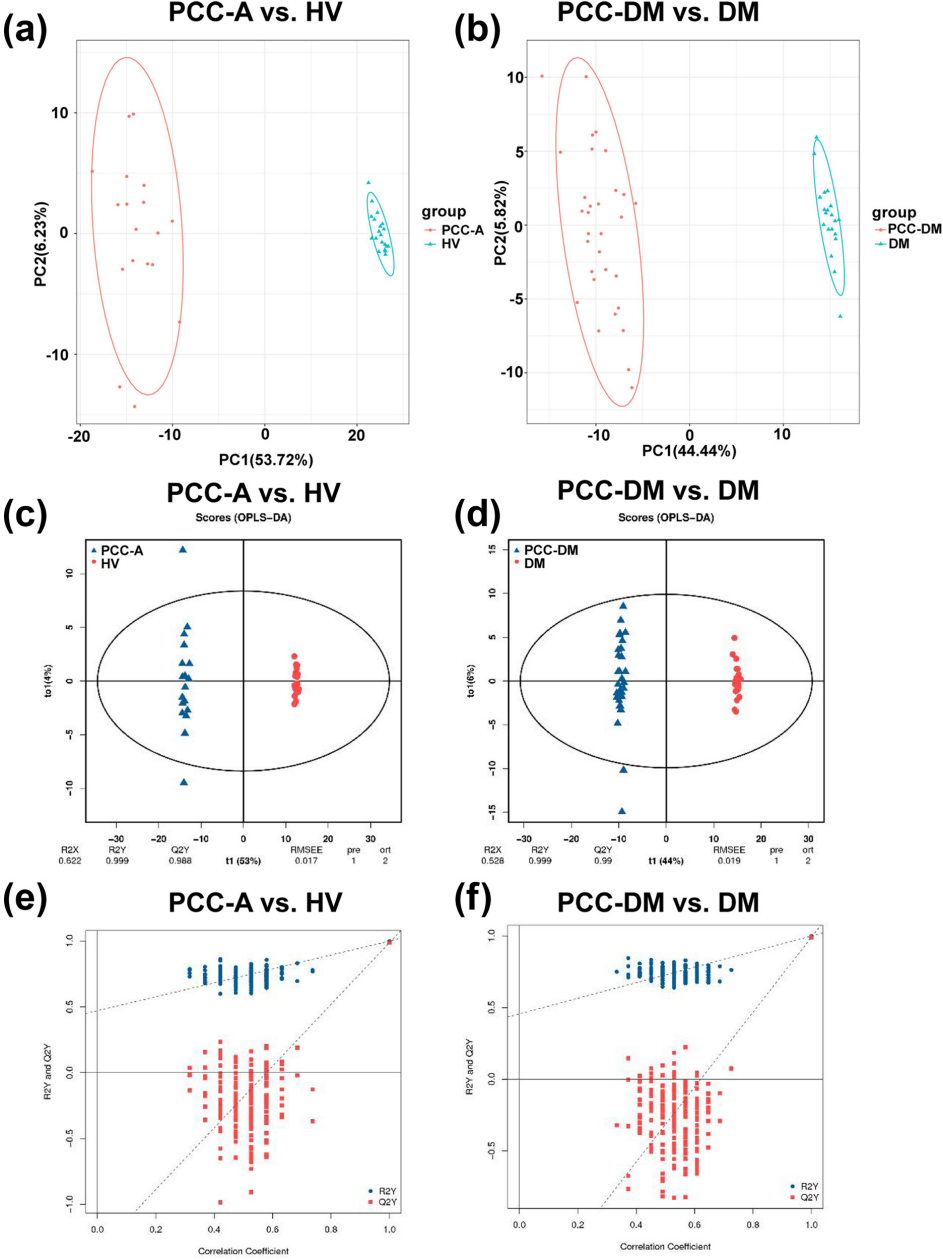
Multivariate analysis of metabolites from patients with PCC-A, patients with PCC-DM, and corresponding controls. (a and b) PCA score plot based on the metabolites in PCC-A vs HV groups and PCC-DM vs DM groups. (c and d) OPLS-DA plot based on the metabolites in PCC-A vs HV groups and PCC-DM vs DM groups. (e and f) OPLS-DA permutation test in PCC-A vs HV groups and PCC-DM vs DM groups. PCC-A: PCC alone. HV: healthy volunteer. PCC-DM: PCC combined with type 2 diabetes mellitus. DM: type 2 diabetes mellitus.

PCA and OPLS-DA were utilized to evaluate the difference in plasma metabolites in B vs A. The correlation analysis showed that the correlation coefficients within groups were higher than those between groups, indicating that the identified differential metabolites are reliable (Figure 1a). The PCA score plot showed that the percentage of principal components 1 and 2 were 16.47 and 6.71%, respectively (Figure 1b). OPLS-DA analysis revealed a significant separation between the PCC-A and PCC-DM groups of plasma samples (Figure 1c). Additionally, the model exhibited a satisfactory fit, as indicated by R2Y = 0.817 and Q2Y = 0.418 (Figure 1c). The OPLS-DA permutation validation revealed that the original R2 and Q2 parameters for the two groups surpassed the respective values post-*Y*-axis permutation, signifying that the model exhibits an appropriate fit (Figure 1d). Similarly, PCA and OPLS-DA were conducted in PCC-A vs HV and PCC-DM vs DM. The PCA score plot revealed a clear distinction in the metabolic profiles between the PCC-A and HV groups, as well as between the PCC-DM and DM groups (Figure 2a and b). Concurrently, the OPLS-DA model demonstrated a significant isolation between the PCC-A and HV groups as well as the PCC-A and DM groups of plasma samples (Figure 2c and d). The model exhibited a robust fit, with R2Y = 0.999 and Q2Y = 0.988 in PCC-A vs HV groups, and R2Y = 0.999 and Q2Y = 0.99 in PCC-DM vs DM groups. The OPLS-DA permutation test confirmed that the original R2 and Q2 values for PCC – PCC-A vs HV groups and PCC-DM vs DM groups exceeded those obtained after *Y*-axis permutation (Figure 2e and f). Overall, the metabolic profiles of PCC patients exhibit significant alterations.

### 3.3 Identification of differential nucleic acid metabolites

**Figure 3:**
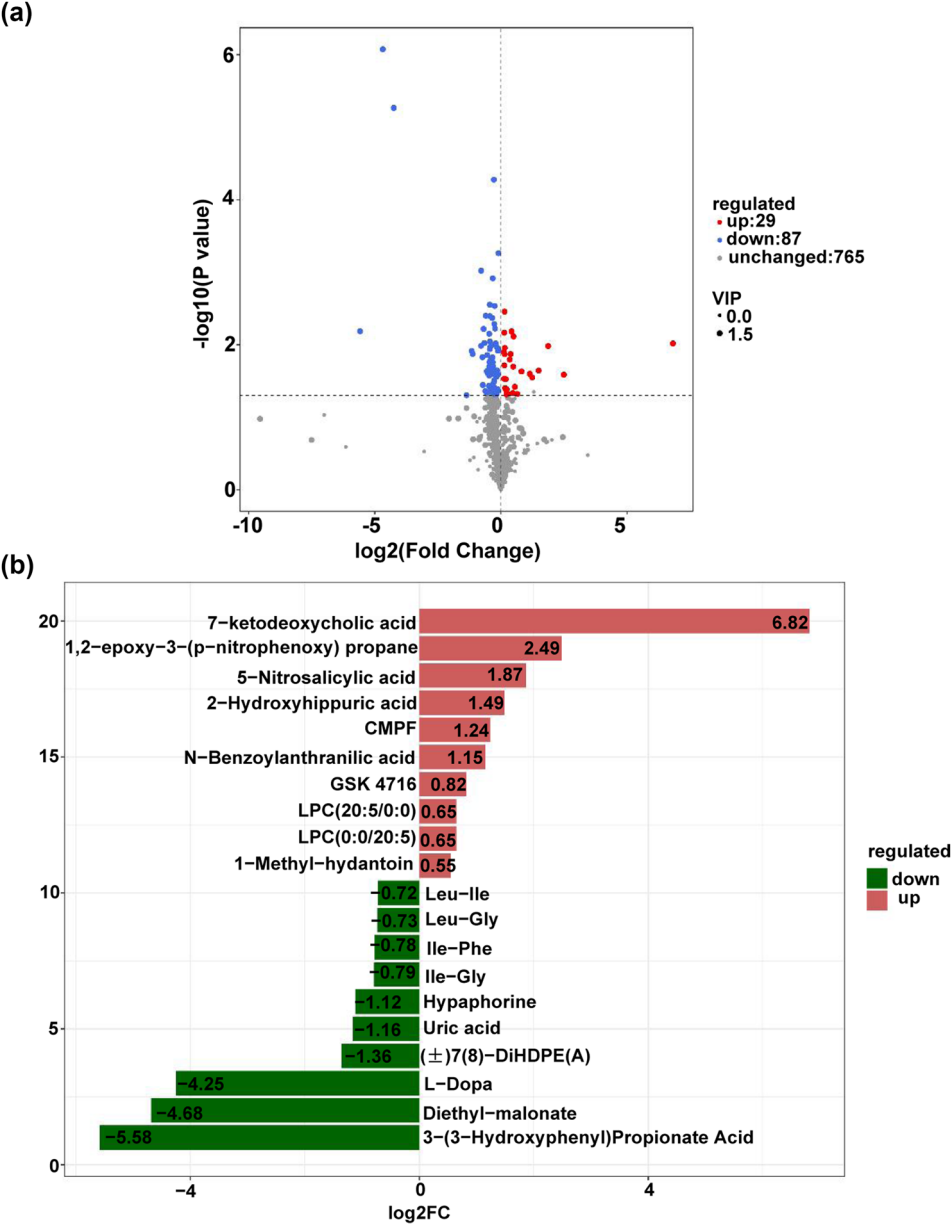
Identification of differential metabolites between PCC-A and PCC-DM. (a) The volcano plot of the plasma metabolomics between the PCC-DM and PCC-A groups. (b) The top 10 upregulated and downregulated metabolites in the PCC-DM group compared with the PCC-A group. PCC-A: PCC alone. PCC-DM: PCC combined with type 2 diabetes mellitus.

**Figure 4:**
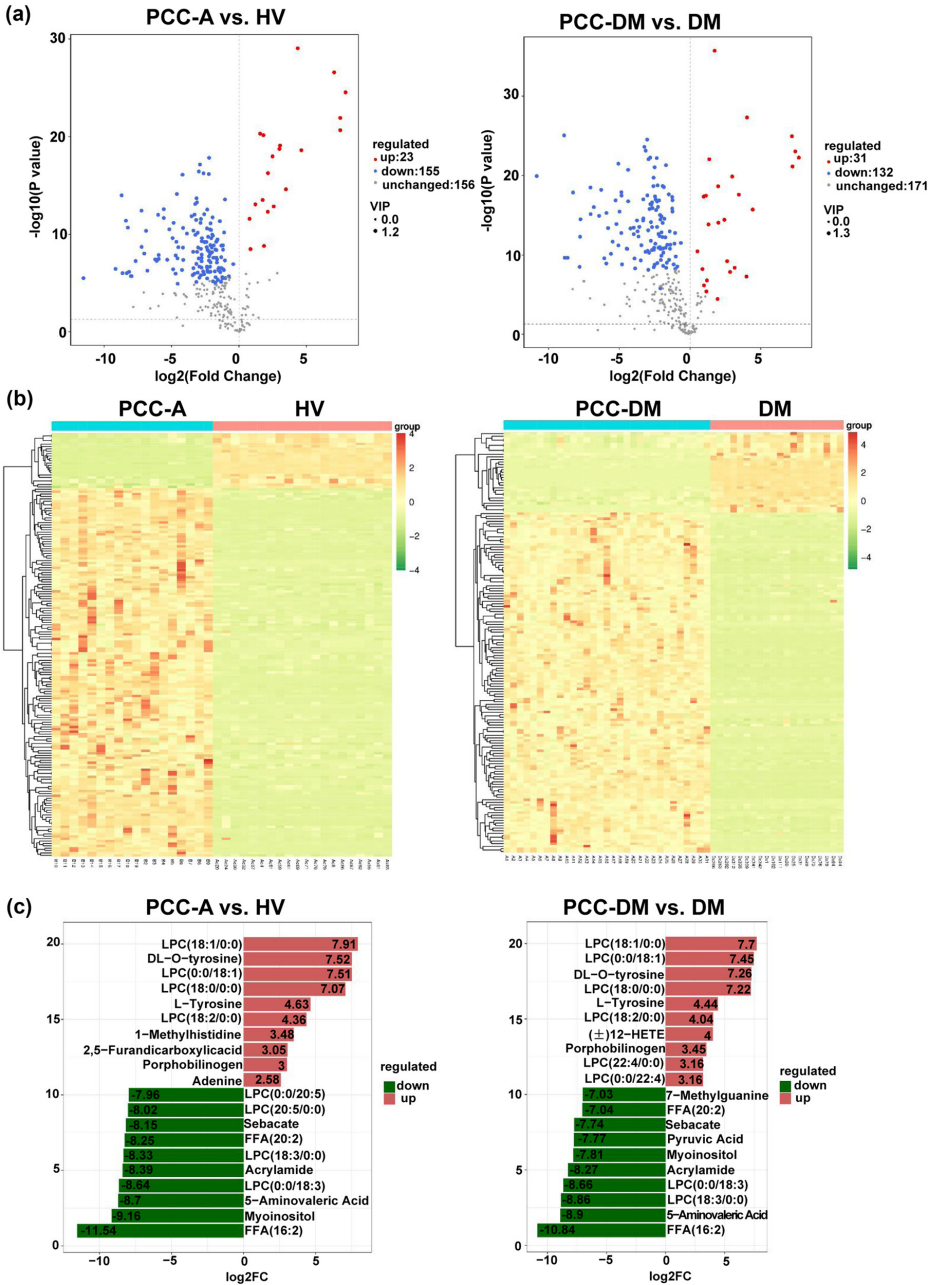
Identification of differential metabolites between PCC-A, PCC-DM, and corresponding controls. (a) The volcano plot of the plasma metabolomics in PCC-A vs HV groups and PCC-DM vs DM groups. (b) Heatmap of differential metabolites of PCC-A vs HV groups and PCC-DM vs DM groups. (c) The top 10 upregulated and downregulated metabolites in PCC-A vs HV groups and PCC-DM vs DM groups. PCC-A: PCC alone. HV: healthy volunteer. PCC-DM: PCC combined with type 2 diabetes mellitus. DM: type 2 diabetes mellitus.

Differential metabolites were identified based on the criteria of VIP greater than 1 and *p*-values less than 0.05. Compared to the group PCC-A, group PCC-DM identified 116 differential metabolites, comprising 29 upregulated and 87 downregulated metabolites (Figure 3a). Figure 3b illustrates the top 10 upregulated and downregulated metabolites in the PCC-DM group as compared to the PCC-A group. Hypaphorine, uric acid, (±)7(8)-DiHDPE(A), and diethyl-malonate were significantly decreased, whereas 7-ketodeoxycholic acid was remarkably increased in the PCC-DM group compared with the PCC-A group. Additionally, a total of 178 and 163 differential metabolites were identified in PCC-A vs HV groups and PCC-DM vs DM groups, respectively (Figure 4a). Compared with the HV group, 155 metabolites were upregulated in the PCC-A group, including l-cystine and uracil, while 23 were downregulated, including l-tyrosine, l-phenylalanine, and adenine (Table S4). Compared with the DM group, 132 metabolites were upregulated in the PCC-DM group, including l-glutamine, 6-*O*-methylguanine, 1-methylguanine, and uracil, while 31 were downregulated, including adenine and l-tyrosine (Table S5). The heatmap revealed a distinct classification of the metabolomes between the PCC-A and HV groups, as well as between the PCC-DM and DM groups (Figure 4b). Compared with the HV group, 1-methylhistidine, l-tyrosine, and adenine were remarkably decreased, whereas 5-acrylamide and sebacate were obviously increased in the PCC-A group (Figure 4c). Compared with the DM group, l-tyrosine and porphobilinogen were remarkably decreased, whereas 7-methylguanine, sebacate, and pyruvic acid were obviously increased in the PCC-DM group (Figure 4c).

**Figure 5:**
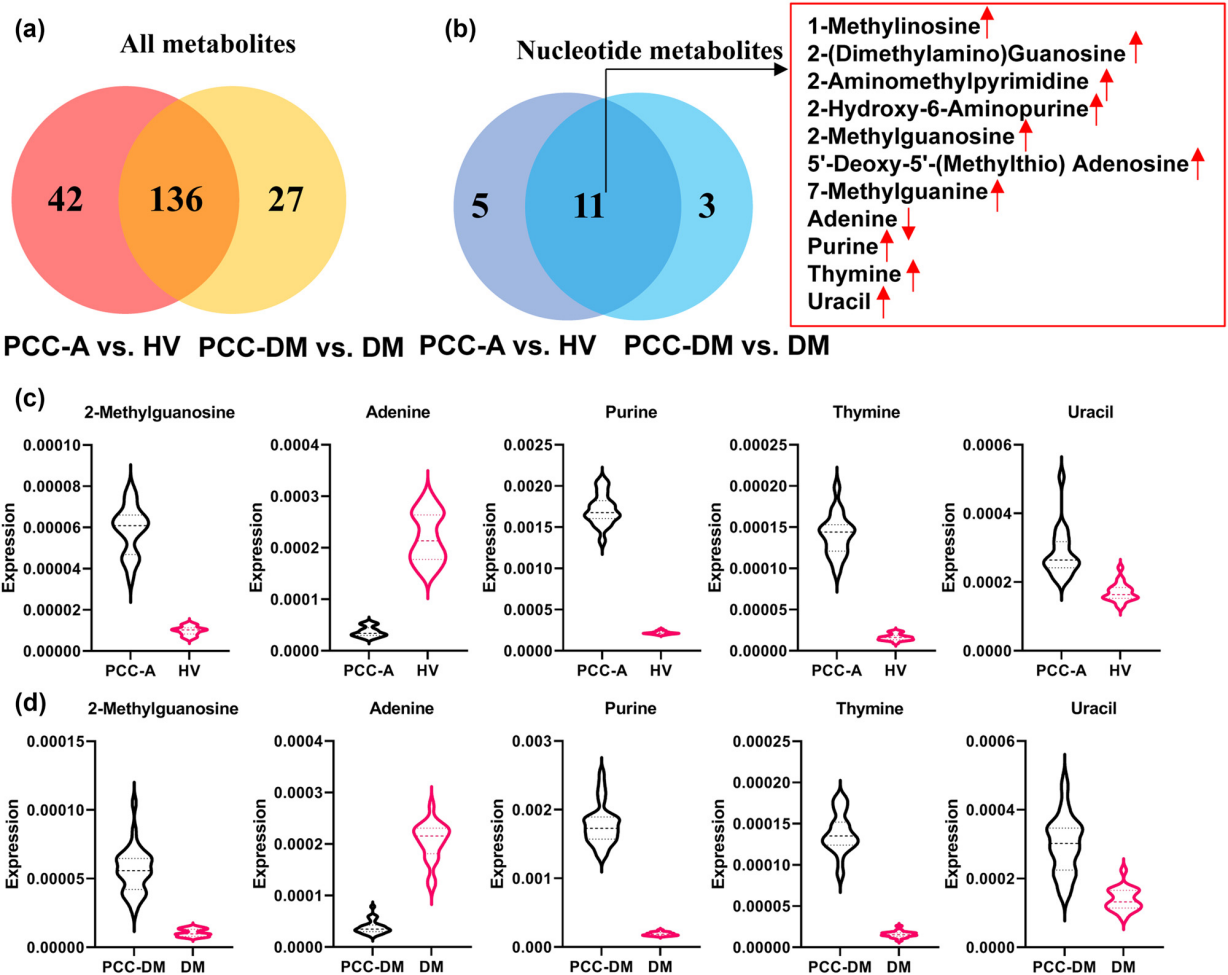
Analysis of differential metabolites related to nucleotide metabolism. (a) Venn diagram of common differential metabolites between PCC-A vs HV groups and PCC-DM vs DM groups. (b) Venn diagram of common differential metabolites related to nucleotide metabolism between PCC-A vs HV groups and PCC-DM vs DM groups. (c) The expression of metabolites related to nucleotide metabolism in PCC-A and HV groups. (d) The expression of metabolites related to nucleotide metabolism in PCC-DM and DM groups.

Subsequently, the common differential metabolites between the PCC-A vs HV group and the PCC-DM vs DM group were analyzed. As shown in Figure 5a, 136 common differential metabolites were identified between the PCC-A vs HV group and the PCC-DM vs DM group. Among these metabolites, we identified 11 common differential metabolites related to nucleotide metabolism between the PCC-A vs HV group and the PCC-DM vs DM group (Figure 5b). The expression of 2-methylguanosine, purine, thymine, and uracil was remarkably enhanced, while adenine was diminished in the PCC-A group compared with the HV group and in the PCC-DM group compared with the DM group (Figure 5c and d). Overall, abnormal nucleotide metabolism occurred in PCC patients.

### 3.4 Analysis of pathways associated with nucleotide metabolism

**Figure 6:**
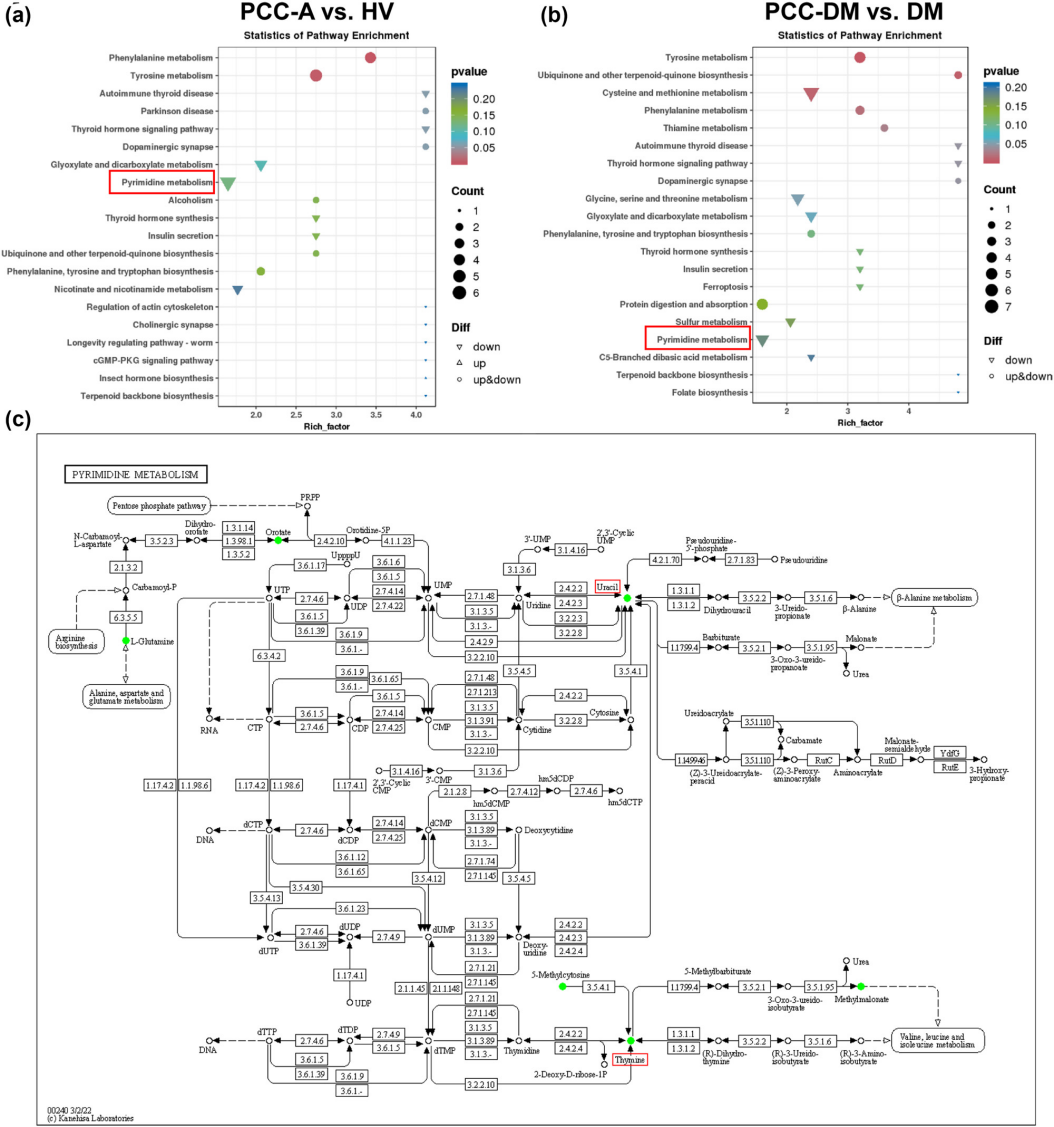
Metabolic pathway enrichment analysis. Metabolic pathway enrichment analysis plot revealed remarkably changed pathways in PCC-A vs HV groups (a) and PCC-DM vs DM groups (b). (c) Pyrimidine metabolism. PCC-A: PCC alone. HV: healthy volunteer. PCC-DM: PCC combined with type 2 diabetes mellitus. DM: type 2 diabetes mellitus.

To elucidate the metabolic characteristics of PCC patients, we conducted a metabolic pathway enrichment analysis. Differential metabolites between PCC-DM and PCC-A groups were mainly enriched in glycine, serine, and threonine metabolism, Carbohydrate digestion and absorption, cysteine and methionine metabolism, and ABC transporters (Figure S1). Differential metabolites between PCC-A and HV groups were mainly enriched in tyrosine metabolism, pyrimidine metabolism, and nicotinate and nicotinamide metabolism (Figure 6a). Differential metabolites between the PCC-DM and DM groups were mainly enriched in tyrosine metabolism, cysteine and methionine metabolism, and glycine and pyrimidine metabolism (Figure 6b). As shown in Figure 6c, uracil and thymine were involved in pyrimidine metabolism. Given the involvement of metabolites from both the PCC-A vs HV and PCC-DM vs DM groups in pyrimidine metabolism, we speculate that patients with PCC may experience disorders of nucleotide metabolism.

### 3.5 The relationship between F and ACTH levels and nucleic acid metabolites

To determine the relationship between metabolic abnormalities and ACTH and F levels after stress in PCC patients, we analyzed the correlation between nucleic acid metabolites (adenine, uracil, 6-*O*-methylguanine, 7-methylguanine, 1-methylguanine, and 2-deoxyribose 1-phosphate) and F and ACTH levels. The expression of adenine was positively correlated with the growth value of F secretion at 90 min after arginine stimulation test (Table 4). However, there was no correlation between F and ACTH growth values and uracil, 6-*O*-methylguanine, 7-methylguanine, 1-methylguanine, and 2-deoxyribose 1-phosphate.

**Table 4:** The relationship between F and ACTH levels and nucleic acid metabolites in PCC patients undergoing the arginine stimulation test

Metabolites		F 0 min growth value (nmol/L)	F 30 min growth value (nmol/L)	F 60 min growth value (nmol/L)	F 90 min growth value (nmol/L)	ACTH 0 min growth value (pmol/L)	ACTH 30 min growth value (pmol/L)	ACTH 60 min growth value (pmol/L)	ACTH 90 min growth value (pmol/L)
Adenine	r	0.162	0.178	0.274	0.328	0.177	0.207	0.256	0.238
	p	0.265	0.221	0.057	0.022	0.224	0.153	0.076	0.099
Uracil	r	−0.033	−0.074	−0.041	0.027	−0.184	−0.077	0.02	0.067
	p	0.822	0.615	0.778	0.852	0.205	0.598	0.89	0.646
6-*O*-Methylguanine	r	−0.156	−0.043	0.016	0.068	−0.126	0.041	0.071	0.125
	p	0.284	0.772	0.913	0.643	0.387	0.78	0.628	0.394
7-Methylguanine	r	−0.156	−0.043	0.016	0.068	−0.126	0.041	0.071	0.125
	p	0.284	0.772	0.913	0.643	0.387	0.78	0.628	0.394
1-Methylguanine	r	−0.156	−0.043	0.016	0.068	−0.126	0.041	0.071	0.125
	p	0.284	0.772	0.913	0.643	0.387	0.78	0.628	0.394
2-Deoxyribose 1-phosphate	r	−0.018	−0.018	−0.03	−0.051	−0.033	−0.145	−0.139	−0.201
	P	0.903	0.902	0.838	0.73	0.824	0.319	0.34	0.166

### 3.6 Biomarkers identification

**Figure 7:**
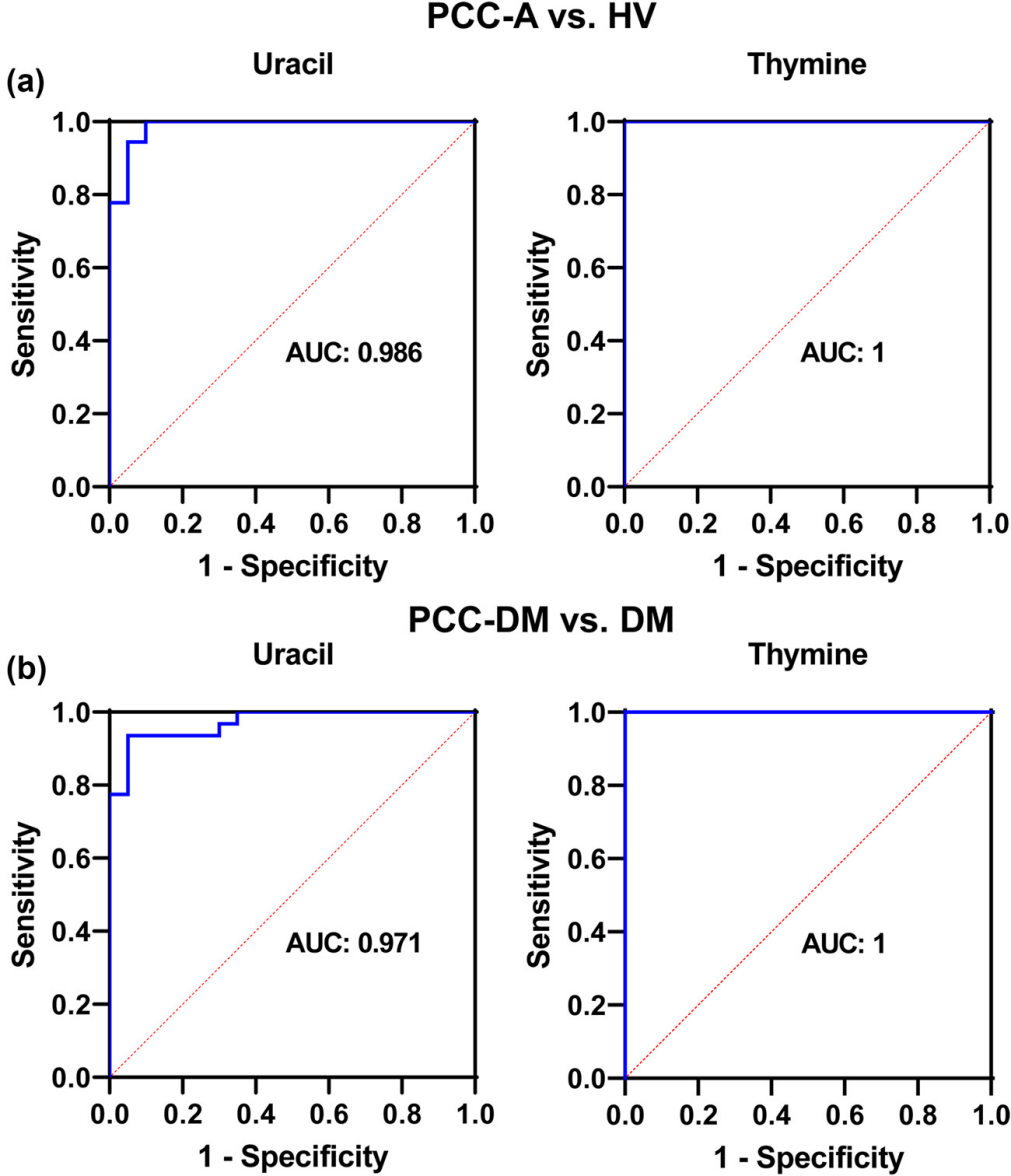
ROC curve analysis of the diagnostic values of metabolites in PCC patients. (a) Biomarkers identified in PCC-A vs HV groups. (b) Biomarkers identified in the PCC-DM vs DM groups. PCC-A: PCC alone. HV: healthy volunteer. PCC-DM: PCC combined with type 2 diabetes mellitus. DM: type 2 diabetes mellitus.

Receiver operating characteristic (ROC) curve analysis was used to identify biomarkers in PCC patients. Uracil and thymine, which are related to pyrimidine metabolism, were analyzed. The results showed that the area under the ROC curves (AUCs) for uracil and thymine in PCC-A vs HV groups were 0.986 and 1, respectively (Figure 7a). The AUCs for uracil and thymine in PCC-DM vs DM groups were 0.971 and 1, respectively (Figure 7b). These results indicated that uracil and thymine had the potential to be biomarkers for PCC patients.

## 4 Discussion

PCC patients had symptoms such as fatigue, chest tightness, joint muscle pain, fatigue, sleep disorders, and psychological abnormalities [30], but routine and biochemical examinations showed no obvious abnormalities. We highly suspected that there was an internal environment disorder at this time. We used UPLC–MS/MS to analyze differential metabolites in PCC patients. Compared with the PCC-A group, 29 differential metabolites were up-regulated and 87 differential metabolites were down-regulated in the PCC-DM group. Further comparison of metabolites with HV and DM patients showed that PCC patients mainly had abnormal nucleotide metabolism.

Nucleotides, including purine nucleotides and pyrimidine nucleotides, are active precursors of nucleic acids and participate in many biological processes in the body as mediators [31]. Nucleotides are mainly synthesized by the body cells themselves, and there are two ways of synthesis: *de novo* synthesis and salvage pathway [32]. The *de novo* synthesis mainly occurs in the liver, and aspartate and glutamine are the main sources of purine and pyrimidine bases [33]. In this study, adenine was significantly down-regulated and uracil was significantly up-regulated in patients with PCC alone compared with normal controls. Compared to patients with DM alone, 6-*O*-methylguanine, 1-methylguanine, 7-methylguanine, and uracil were significantly up-regulated, and adenine was significantly down-regulated in patients with PCC-DM. These findings indicate that PCC patients exhibit abnormal nucleotide metabolism.

The main raw material of the salvage pathway is the free purine/pyrimidine base produced by nucleotide degradation. Because the human brain lacks the enzyme system of *de novo* synthesis of purine nucleotides, the salvage pathway can only be used. Adenine can assist in the synthesis of DNA and RNA and is a key regulatory factor in the maintenance of physiological processes [34,35]. When NAD+ levels are reduced, aging, diabetes, cancer, neurodegeneration, and cardiovascular disease are significantly increased [36]. In this study, abnormal *de novo* synthesis was found in PCC patients, and adenine expression was further reduced in patients with PCC-DM, and uric acid was significantly reduced. These results suggest that both *de novo* nucleotide synthesis and remedial synthesis pathways are seriously damaged in PCC patients, especially in patients with PCC-DM.

The diagnosis and treatment of PCC remain challenging, and there is a lack of biomarkers for early recognition and intervention. In recent years, significant progress has been made in the study of biomarkers for PCC patients. Gu et al. identified 23 potential biomarkers that could influence the long-term consequences of COVID-19, which could help identify patients with PCC at high risk [37]. Cervia-Hasler et al. discovered that patients with PCC showed increased markers of hemolysis, tissue damage, platelet activation, and monocyte–platelet aggregates and confirmed complement and thromboinflammatory proteins as PCC biomarkers [38]. In this study, we preliminarily identified uracil and thymine as potential biomarkers for PCC patients using ROC curves. Uracil and thymine are important components of nucleotides involved in the synthesis and metabolism of DNA and RNA. In patients with PCC, abnormal levels of these two substances may reflect disruption of nucleotide metabolism in the body. However, it is important to note that current research on the role of uracil and thymine in PCC is still in its preliminary stages. Future studies require larger-scale clinical trials to validate these findings and to further explore their interactions with other pathological mechanisms of PCC. Additionally, the expression of these biomarkers may vary among different patient groups, highlighting the need for personalized diagnosis and treatment strategies as a crucial direction for future research.

Studies have confirmed that COVID-19 can invade many parts of the brain, including the hypothalamus and pituitary gland [8]. Among patients who underwent low-dose ACTH stimulation tests 3 months after COVID-19 infection, 16.2% showed insufficient F response [39,40]. In this study, we performed arginine stimulation tests on PCC patients, and PCC patients showed a decreased trend of ACTH and F, which may be related to multiple endocrine axis dysfunction caused by the COVID-19 virus attack on the ventromedial nucleus of the hypothalamus. The hypothalamus–pituitary–adrenal (HPA) axis is composed of the hypothalamus, pituitary gland, and adrenal gland and is the core of homeostasis, stress response, energy metabolism, and neuropsychiatric function [41]. F is a hormone secreted by the adrenal gland, which plays an important role in the regulation of metabolism, immunity, and blood pressure [42]. The decrease of F may lead to fatigue, low blood pressure, low blood sugar, and decreased immunity [43]. The deficiency of ACTH and F can be life-threatening under stress conditions (such as infection, surgery, and trauma) [44]. For patients with PCC, a direct consequence of low F levels is a decrease in the brain’s ability to suppress inflammation [45]. When the patient encounters the stressor again, the inflammatory response of the brain may become extremely intense and even lead to repeated episodes of symptoms. Normally, low cortisol levels should be compensated for by an increase in corticotropin produced by the pituitary gland. However, no such compensatory increase was observed in patients with PCC, indicating dysfunction of the HPA axis [46]. However, the underlying mechanisms of ACTH and F changes remain to be further investigated. Furthermore, we found that adenine was positively correlated with F growth value at 90 min after the arginine stimulation test. Together, there may be a complex interaction between ACTH and F levels and the disturbance of nucleotide metabolism in patients with PCC. Future studies need to further clarify the specific mechanism of this relationship to provide new ideas and targets for the diagnosis and treatment of PCC.

This study has some limitations in exploring the abnormal nucleotide metabolism and its relationship with endocrine function in patients with PCC. First, due to the limited scope of the metabolite panel, we may not have covered all important nucleotide metabolites. Future research needs to expand the scope of the metabolite panel to include a more diverse range of nucleotide metabolites in order to obtain more comprehensive and accurate research conclusions. Second, this study primarily relied on metabolomic analysis and hormone level analysis, failing to deeply investigate the complex interactions between nucleotide metabolism and the endocrine system. Finally, as PCC is an emerging disease and related research is still in its infancy, this study may not have fully leveraged and integrated existing knowledge, potentially leading to gaps in understanding. Therefore, future research needs to expand the sample size, employ interdisciplinary approaches, and consider more potential influencing factors to further uncover the nature of the relationship between nucleotide metabolism abnormalities and the endocrine system in patients with PCC.

In summary, the *de novo* synthesis and remedial synthesis pathways in patients with PCC, especially in patients with PCC-DM, are seriously damaged, leading to the disorder of the body’s cell metabolic pathways and the imbalance of cell metabolism. The abnormal secretion of ACTH and F after stress may be an important reason for the dysfunction of nucleotide metabolism.

## Abbreviations

ACTH: adrenocorticotropic hormone
ALB: albumin
ALT: alanine aminotransferase
AST: aspartate aminotransferase
COVID-19: coronavirus disease 2019
Cr: creatinine
DM: diabetes mellitus
F: cortisol
GC–MS: gas chromatography–mass spectrometry
GSI: nebulizer gas
GSII: heater gas
HDL: high-density lipoprotein
HV: healthy volunteers
LC–MS: liquid chromatography–mass spectrometry
LDL: low-density lipoprotein
OPLS-DA: orthogonal partial least squares discriminant analysis
PCC: post-COVID-19 condition
PCC-A: PCC alone
PCC-DM: PCC combined with type 2 diabetes mellitus
TC: total cholesterol
TG: triglyceride
TP: total protein
UA: uric acid
UPLC–MS/MS: ultraperformance liquid chromatography–mass spectrometry
VIP: variable importance in projection

## Supplementary Material

Supplementary Figure

Supplementary Table 1

Supplementary Table 2

Supplementary Table 3

Supplementary Table 4

Supplementary Table 5
