# Supplementary material for: Nucleotide metabolic abnormalities in post-COVID-19 condition and type 2 diabetes mellitus patients and their association with endocrine dysfunction
Source: Open Med (Wars). 2025 Oct 7;20(1):20251221. doi: 10.1515/med-2025-1221 (PMC12514782; doi:10.1515/med-2025-1221)
Supplement: Supplementary Figure [file med-2025-1221-sm.pdf]

Supplementary material

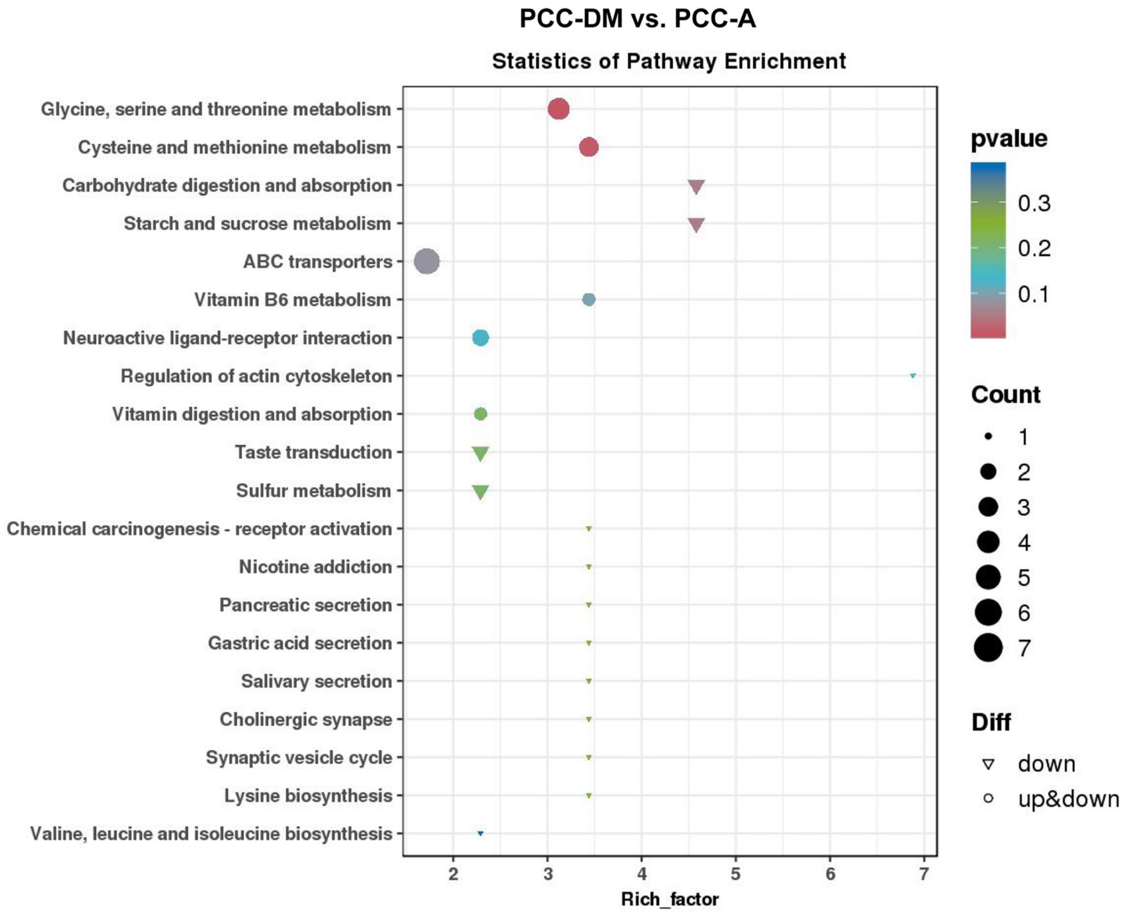

Figure S1: Metabolic pathway enrichment analysis in PCC-DM vs PCC-A groups. PCC-DM: PCC combined with type 2 diabetes mellitus. DM: type 2 diabetes mellitus.
